# Self-management behaviors in patients with H-type hypertension: a latent profile analysis within the health ecological model

**DOI:** 10.3389/fcvm.2026.1903891

**Published:** 2026-10-21

**Authors:** Lei Ma, Jingru Liang, Xiuqing Fang, Mengjie Yang, Hong Zhang, Tong Zhao, Ting Liu

**Affiliations:** 1 Health Management Center, The Affiliated Hospital of Qingdao University, Qingdao, China; 2 School of Nursing, Qingdao University, Qingdao, China

**Keywords:** health behavior, hyperhomocysteinemia, hypertension, latent profile analysis, self-management

## Abstract

**Background:**

H-type hypertension confers a substantially greater risk of stroke than elevated blood pressure or homocysteine alone. Beyond stroke, hyperhomocysteinemia is implicated in endothelial dysfunction, vascular oxidative stress, arterial stiffening, coronary artery disease, heart failure, and progression of chronic kidney disease. Self-management is an effective secondary prevention strategy for H-type hypertension, yet research on self-management behaviors in these patients remains limited and has mostly adopted a variable-centered approach.

**Objective:**

The study aimed to explore the current status and latent profiles of self-management behaviors among patients with H-type hypertension, and to examine the factors associated with profile membership within the health ecological model.

**Method:**

Convenience sampling was used to recruit 310 patients from a tertiary hospital in eastern China. Data were collected using a general information questionnaire and standardized instruments assessing hypertension self-management behaviors, eHealth literacy, perceived social support, perceived stress, hospital anxiety and depression, and resilience. Mplus 8.3 and SPSS 26.0 were used to identify latent profiles and the correlates of profile membership.

**Results:**

The mean standardized self-management behavior score was 67.34 ± 13.82. Four latent profiles of self-management behaviors were identified: Low self-management pattern (18.4%), Moderate self-management-treatment monitoring pattern (21.0%), Moderate self-management-lifestyle pattern (19.0%), and High self-management pattern (41.6%). Profile membership was significantly associated with the presence of comorbidity, eHealth literacy, and social support.

**Conclusions:**

Self-management behaviors among patients with H-type hypertension were at a moderate level and showed substantial heterogeneity, with four distinct profiles. Individualized strategies tailored to the characteristics of each profile, targeting potentially modifiable correlates such as eHealth literacy and perceived social support, may be worth developing and evaluating prospectively in order to enhance patients’ self-management behaviors.

## 1 Introduction

According to the 2019 Global Burden of Disease (GBD), stroke is the second leading cause of death worldwide and the third leading cause of disability-adjusted life years (DALYs), imposing a significant burden on global public health systems and human health. H-type hypertension, defined as hypertension accompanied by elevated homocysteine (Hcy ≥ 10 μmol/L) (1), is an important risk factor contributing to the high prevalence of stroke. A cohort study on chronic diseases revealed that in the Chinese population, an isolated increase in blood pressure can raise the risk of stroke by 9.7 times, while an isolated increase in Hcy levels (≥10 μmol/L) can raise the risk of stroke by 3.5 times. However, hypertension combined with high Hcy can elevate the risk of stroke and stroke-related death by 12.7 times and 11.7 times, respectively (1). This evidence clearly indicates that H-type hypertension poses a far greater threat to stroke incidence and mortality than either high blood pressure or Hcy alone. The cardiovascular relevance of hyperhomocysteinemia is not, however, confined to cerebrovascular events. Elevated Hcy may contribute to endothelial dysfunction and vascular oxidative stress, thereby promoting vascular remodeling and arterial stiffening. Clinically, elevated Hcy levels have been associated with coronary artery disease, adverse cardiac remodeling and progression of chronic kidney disease (2–6). Patients with H-type hypertension therefore carry a compounded burden of target-organ damage across the whole cardiovascular continuum, which further underlines the clinical importance of sustained, patient-led disease management in this population. Research has shown that H-type hypertension accounts for 73.1% of hypertensive patients in China (7). Thus, effective control of H-type hypertension has become a critical component in stroke prevention and treatment in China. Self-management serves as an effective secondary prevention strategy for hypertension (8). Therefore, investigating the current status of and the factors associated with self-management behaviors among individuals with H-type hypertension holds significant practical and public health value for promoting self-management in this population and reducing the stroke burden in China.

Studies on self-management behaviors in patients with H-type hypertension are rare, and most employ a variable-centered approach that uses the total self-management score as a whole (9, 10). This method fails to capture variations in self-management behaviors across individuals, thereby limiting the effectiveness of relevant interventions developed on the basis of its findings. Latent profile analysis (LPA) is a person-centered approach that identifies distinct subgroups of individuals based on patterns of observed characteristics and thereby characterizes heterogeneity within a population (11, 12). In the present study, LPA was used to identify distinct profiles of self-management behaviors among patients with H-type hypertension, and factors associated with profile membership were subsequently examined. In this way, LPA can assist in formulating more precise interventions tailored to the specific characteristics of each profile (13). Compared with conventional variable-centered approaches, person-centered approaches provide a complementary perspective by focusing on patterns of characteristics within individuals rather than primarily on associations among variables at the population level (12, 14). Variable-centered models may obscure subgroups characterized by distinct combinations of behavioral strengths and deficits, whereas person-centered approaches such as LPA identify relatively homogeneous subgroups based on multivariate response patterns across multiple domains (12, 14). Thus, LPA can differentiate patients who share similar total scores but exhibit opposite strengths and deficits (e.g., poor medication adherence but adequate diet vs. the reverse), allowing interventions to be matched to specific profiles (13, 15). The resulting subgroup classification could directly inform stratified follow-up content and intensity in cardiovascular care research.

The Health Ecological Model posits that health outcomes are shaped by the interaction of multiple factors across different levels, including individual characteristics, behavioral patterns, interpersonal networks, living and working conditions, and broader policy and environmental contexts (16, 17). This framework highlights the multidimensional and complex determinants of health. Such a multilevel perspective is particularly valuable in the context of chronic diseases, which are characterized by complex and long-term interactions among biological, behavioral, and environmental factors (18, 19). Evidence indicates that effective self-management in chronic conditions is associated not only with individual clinical characteristics but also with behavioral, psychological, and social factors (20). For instance, unhealthy lifestyle behaviors such as smoking, alcohol consumption, and poor sleep are consistently associated with worse disease control and prognosis (21). In addition, psychological factors, including anxiety, depression, and resilience, are closely associated with patients’ adherence to treatment and engagement in self-management behaviors (22–24). Beyond individual and behavioral factors, interpersonal and social contexts are also crucial. Social support, educational attainment, and marital status have been consistently associated with health behaviors and outcomes in chronic disease populations (25). Moreover, structural determinants, such as socioeconomic status, access to healthcare services, and insurance coverage, are consistently associated with healthcare utilization and long-term disease management (26, 27). Together, these findings highlight the necessity of adopting a multilevel framework to comprehensively understand self-management behaviors in patients with chronic diseases. This is particularly true in patients with H-type hypertension, a subtype characterized by the coexistence of hypertension and elevated homocysteine levels, where the interaction between metabolic abnormalities and lifestyle-related risk factors further amplifies the complexity of disease management (4). Applying a multilevel ecological framework is therefore essential to capture the heterogeneity of self-management behaviors in this population. This study aimed to identify heterogeneous self-management patterns among patients with H-type hypertension by using LPA and to examine the multilevel factors associated with profile membership. By mapping the available variables onto the five ecological levels, the findings will offer a structured perspective on the multidimensional factors associated with self-management behaviors and may inform targeted and personalized intervention strategies.

## 2 Methods

### 2.1 Participants and recruitment

The study used a cross-sectional design. Patients with H-type hypertension were recruited by convenience sampling from the outpatient department and health examination center of a general tertiary hospital in Shandong Province. The inclusion criteria for participants were (a) aged ≥18 years, (b) diagnosed with H-type hypertension (hypertension accompanied by Hcy ≥ 10 μmol/L) (1), (c) with disease duration ≥6 months, and (d) willing to participate in the study voluntarily and provide written informed consent. The exclusion criteria were (a) a diagnosis of secondary hypertension, and (b) the presence of serious comorbidities, defined as New York Heart Association (NYHA) functional class III–IV heart failure (28), acute coronary syndrome within the preceding six months, stroke with residual functional dependence (defined as a modified Rankin Scale score ≥ 3) (29), chronic kidney disease of stage 4 or above (estimated glomerular filtration rate <30 mL/min/1.73m^2^) (30) or receipt of dialysis, chronic obstructive pulmonary disease requiring long-term oxygen therapy, or active malignancy. These conditions were excluded because they often impair patients’ functional capacity to perform the exercise, dietary, and self-monitoring behaviors assessed by the self-management instrument in the study.

Specifically, hypertension was diagnosed by the treating physician according to the *Chinese Guidelines for the Prevention and Treatment of Hypertension (2018 revision)* (31), defined as systolic blood pressure ≥140 mmHg and/or diastolic blood pressure ≥90 mmHg on at least two separate occasions, or a self-reported history of physician-diagnosed hypertension with current use of antihypertensive medication. H-type hypertension was defined as the coexistence of essential hypertension and elevated plasma homocysteine (Hcy) ≥10 μmol/L (1). At enrollment, all participants underwent standardized fasting venous blood sampling for Hcy measurement. Patients already receiving antihypertensive treatment continued their existing regimens. Those taking folic acid or B-vitamin supplements at enrollment were not excluded. However, current use of these agents was recorded in the general information questionnaire.

From June 2024 to December 2024, a total of 323 paper-based questionnaires were distributed and returned. After excluding 13 questionnaires due to incomplete responses (*n* = 6, 1.86%) or patterned answering (*n* = 7, 2.17%), 310 valid questionnaires were retained for analysis, representing a valid questionnaire proportion of 95.97%. Patterned answering was defined as straight-line responses (i.e., selecting the same option across all items within a subscale or across the entire questionnaire) or discernible zigzag/alternating patterns. The 310 retained questionnaires had complete data on all variables used in the analyses. Accordingly, a complete-case analysis was performed, and no imputation or other missing-data handling was required. Sample size requirements for LPA are not determined by a single fixed rule but depend on several model and data characteristics, including the number of indicators, degree of class separation, number of latent classes, and relative class sizes (12). In a Monte Carlo simulation study, Tein et al. (32) demonstrated that class separation was a stronger determinant of statistical power in LPA than sample size, with the number of indicators also contributing to power under some conditions. Critically, they found that with a very large class separation (Cohen's *d* = 1.5), a sample size of 250 was sufficient to achieve adequate power. Additionally, a commonly cited rule of thumb suggests 10–20 cases per indicator for LPA. With six indicator dimensions in the model, this rule would require 60–120 cases. Collectively, these considerations support the adequacy of the sample size for the LPA conducted in this study.

### 2.2 Measures

#### 2.2.1 General information questionnaire

The questionnaire was designed by the researchers to collect sociodemographic information, lifestyle habits, and disease-related information from patients with H-type hypertension. Specifically, the questionnaire consisted of three parts: (a) sociodemographic information, including age, gender, marital status, educational level, occupation status, monthly income, residence, main medical institution for treatment, and type of medical insurance; (b) lifestyle habits, including smoking and drinking. Smoking was defined according to the World Health Organization (WHO) as continuous or cumulative smoking for six months or more during one's lifetime. Alcohol consumption was classified based on the Chinese Dietary Guidelines (2016) (33), where moderate alcohol consumption refers to an average daily alcohol intake not exceeding the recommended amount (25 grams for men, 15 grams for women), and excessive drinking refers to an average daily alcohol intake exceeding the recommended amount; and (c) disease-related information, including duration of hypertension, classification of hypertension, comorbidity, and current use of B vitamins or folic acid tablets. Comorbidity was ascertained from patients’ self-reported history of physician-diagnosed chronic conditions, cross-checked against the outpatient medical records and the laboratory results available at the recruitment visit. Conditions counted as comorbidities included diabetes mellitus, dyslipidemia, coronary heart disease without a recent acute event, cerebrovascular disease without residual functional dependence, and chronic pulmonary disease not requiring long-term oxygen therapy, all of which were considered milder, well-compensated conditions because patients with severe comorbidities had been excluded at recruitment. Comorbidity was operationalized as a binary variable indicating the presence of at least one such condition, rather than as a count of conditions.

#### 2.2.2 Physical and laboratory measurements

Professionally trained staff conducted the physical examinations, including height, weight, body mass index (BMI), and blood pressure. Height and weight were measured twice consecutively using an Omron ultrasonic health examination device, with participants barefoot and wearing light clothing. The average of the two measurements was recorded as the final value. BMI was calculated as weight (kg)/height (m)^2^. Based on the Chinese adult BMI criteria, a BMI of 18.5 to <24.0 kg/m^2^ is considered normal, 24.0 to <28.0 kg/m^2^ overweight, and ≥28.0 kg/m^2^ obese (34). Blood pressure was measured twice using an Omron electric blood pressure monitor after participants had rested quietly for 5 min, with a 2-min interval between readings. The mean value was recorded as a single baseline assessment at recruitment. This baseline measurement was used for descriptive purposes only and was not included as a covariate in the regression model, because a single measurement cannot define sustained blood pressure control.

For laboratory examination, venous blood samples (5 mL) were collected from participants after a 12-h fast to measure plasma levels of Hcy, fasting blood glucose (FBG), triglycerides (TG), total cholesterol (TC), low-density lipoprotein cholesterol (LDL-C), and high-density lipoprotein cholesterol (HDL-C). According to the Chinese Guidelines for the Prevention and Treatment of Type 2 Diabetes (2017 Edition), an FBG level < 6.10 mmol/L is defined as normal (35). According to the Chinese Guideline for Lipid Management (36), TG ≥ 2.3 mmol/L, TC ≥ 6.2 mmol/L, LDL-C ≥ 4.1 mmol/L, and HDL-C < 1.0 mmol/L are considered abnormal lipid levels.

#### 2.2.3 Ehealth literacy scale

The eHealth literacy scale was developed by Norman and Skinner (37) and translated into Chinese by Guo et al. (38). It consists of eight items within three dimensions: ability to use online health information and services, ability to judge online health information, and ability to make decisions. The scale uses a 5-point Likert scoring method, with responses ranging from 1 (“very inconsistent”) to 5 (“very consistent”). The total score ranges from 8 to 40 points, with a high score indicating a high level of eHealth literacy. The Cronbach's *α* coefficient of this scale in this study was 0.904.

#### 2.2.4 Perceived social support scale

This scale was developed by Zimet et al. (39) and was translated into Chinese (40). It consists of 12 items across three dimensions: family support, friend support, and other support. Responses were scored on a 7-point Likert scale, ranging from 1 (“strongly disagree”) to 7 (“strongly agree”). The total score ranges from 12 to 84, with a higher score reflecting the individual's greater perceived level of social support. The Cronbach's *α* coefficient of this scale in this study was 0.876.

#### 2.2.5 Chinese version of perceived stress scale

This scale was used to measure psychological stress (41). It consists of 14 items across two dimensions: perceived stress and perceived coping ability. It is scored on a 5-point Likert scale, ranging from 0 (“never”) to 4 (“always”). The total score ranges from 0 to 56, and a total score ≥26 indicates health-threatening stress. The Cronbach's *α* coefficient of this scale in this study was 0.877.

#### 2.2.6 Hospital anxiety and depression scale

This scale is used to screen for anxiety and depression among patients in general hospitals (42). It consists of 14 items divided into two subscales: anxiety and depression. A total score is calculated for each subscale by summing its respective items. In this study, the Chinese version translated by Ye et al. (43) was adopted, and a higher score indicates a greater tendency toward anxiety or depression. The Cronbach's *α* coefficient of this scale in this study was 0.837.

#### 2.2.7 Brief resilience scale

This scale was developed by Smith et al. (44) to measure an individual's ability to rebound from stress, i.e., psychological resilience. The scale consists of six items scored on a 5-point Likert scale, with a total score ranging from 6 to 30. A higher score indicates a higher level of psychological resilience. The Cronbach's *α* coefficient of this scale in this study was 0.923.

#### 2.2.8 Self-management behavior rating scale for patients with hypertension

This scale was developed based on the self-management theory and consists of 33 items in six dimensions of medication adherence (4 items), self-monitoring (4 items), dietary management (10 items), exercise management (3 items), activity & rest management (5 items), and emotion regulation (7 items) (45). Items are scored on a 5-point Likert scale ranging from 1 (“never”) to 5 (“always”), yielding a total raw score range of 33–165. To describe the overall level of self-management, a standardized overall score was calculated as (actual total raw score/165) × 100, which expresses the score as a percentage of the maximum possible score (theoretical range: 20–100). For descriptive interpretation, this standardized overall score was categorized using thresholds (i.e., <60 was categorized as low, 60–80 as moderate, and >80 as high) reported in a previous study of hypertension self-management (46). For each dimension, a dimension score was calculated as the mean of its constituent items (i.e., the dimension raw score divided by the number of items in that dimension), producing six mean item scores on the original 1–5 scale. These six dimension mean scores served as the indicators for LPA. In this study, the Cronbach's *α* coefficient of this overall scale was 0.924.

### 2.3 Data analysis

Latent profile analysis was performed using Mplus 8.3 software with robust maximum likelihood estimation. The six dimension mean item scores of self-management behaviors were entered as continuous indicators in their original 5-point metric without standardization. Using mean item scores rather than raw summed scores ensured equal weighting across dimensions regardless of the number of items per subscale. All models were specified with class-invariant variances and zero within-class covariances, and this diagonal covariance structure was applied consistently across all models to ensure comparability of fit indices. According to established recommendations, the optimal number of classes was determined by integrating statistical fit indices with theoretical interpretability, class size, and classification certainty. Model fit was evaluated using (a) information criteria: Akaike information criterion (AIC), Bayesian information criterion (BIC), and sample-size-adjusted BIC (aBIC), with lower values indicating better fit; (b) classification evaluation index: entropy, a measure of classification quality ranging from 0 to 1, with values greater than 0.8 indicating good overall classification quality; and (c) the Lo-Mendell-Rubin (LMR) test and the bootstrap likelihood ratio test (BLRT), for which a significant *P* value (<0.05) supports a k-class model over a (*k* − 1)-class model. In addition to these indices, the final model was selected by considering theoretical interpretability, parsimony, profile size, and classification quality (47). Profiles representing approximately 5% or more of the sample were considered preferable for substantive interpretation, and average posterior probabilities of ≥0.80 for each class were considered indicative of good classification accuracy within each profile (48, 49). To avoid convergence on local maxima, a robust random start strategy using 200 random starts with 50 final-stage optimizations was employed. For the final 4-class solution, the best log-likelihood (−2,388.045) was replicated 38 times to confirm solution stability. Descriptive statistical analysis was conducted using SPSS 26.0 software. Measurement data were presented as mean (M) ± standard deviation (SD), and count data as frequencies and percentages. One-way analysis of variance (ANOVA) or chi-square tests were employed to compare across multiple profiles for descriptive purposes only. Because these comparisons were exploratory rather than confirmatory, no formal adjustment for multiplicity was applied. The R3STEP approach was employed to examine factors associated with latent profile membership because it accounts for classification uncertainty by incorporating the misclassification matrix into the multinomial logistic regression. Prior to this analysis, multicollinearity among the candidate variables was assessed using the generalized variance inflation factor (GVIF) to account for the dummy coding of categorical variables. The GVIF was calculated using the vif() function from the car package in R. For categorical variables with multiple degrees of freedom, the adjusted GVIF^[1/(2 × df)]^ was used for interpretation. A *P* value <0.05 was considered statistically significant.

#### 2.3.1 Ethical considerations

This study was approved by the Ethics Committee of the hospital where the study was conducted (approval No: QYFY WZLL 28357). All participants provided informed consent and voluntarily participated in this study.

## 3 Results

### 3.1 Common method bias test

All data in this study were collected using self-report measures. To assess the potential threat of common method bias inherent in self-report data, Harman's single-factor test was conducted as an initial diagnostic check. An unrotated exploratory factor analysis on all measurement items extracted 12 factors, with the first factor accounting for 13.47% of the total variance, which is below the recommended 40% threshold (50), suggesting no dominant single factor.

### 3.2 Demographic characteristics and self-management scores of the participants

The 310 patients were aged between 27 and 85 years, with an average of 57.84 ± 11.19 years. The other demographic information is shown in Table 1. The standardized overall score of self-management behaviors was 67.34 ± 13.82, corresponding to a moderate level according to the grading criteria (46). The average scores of the dimensions of medication adherence, diet management, emotion regulation, activity & rest management, self-monitoring, and exercise management were 3.94 ± 1.46, 3.42 ± 0.83, 3.40 ± 0.95, 3.32 ± 0.93, 3.06 ± 1.00, and 2.77 ± 1.20, respectively.

**Table 1:** General information and univariate analysis of latent profiles of self-management behaviors among patients with H-type hypertension.

Variables	Total (*n* = 310) *n* (%)/M ± SD	Low self-management pattern (*n* = 57) *n* (%)/M ± SD	Moderate self-management-treatment monitoring pattern (*n* = 65) *n* (%)/M ± SD	Moderate self-management-lifestyle pattern (*n* = 59) *n* (%)/M ± SD	High self-management pattern (*n* = 129) *n* (%)/M ± SD	*χ*^2^/*F*	*P*
Age						18.633	**0** **.** **004**
18–44	47 (15.2)	7 (12.3)	6 (9.2)	19 (32.2)	15 (11.6)		
45–59	137 (44.2)	25 (43.8)	29 (44.6)	27 (45.8)	56 (43.4)		
≥60	126 (40.6)	25 (43.9)	30 (46.2)	13 (22.0)	58 (45.0)		
Gender						3.365	0.339
Male	213 (68.7)	37 (64.9)	50 (76.9)	42 (71.2)	84 (65.1)		
Female	97 (31.3)	20 (35.1)	15 (23.1)	17 (28.8)	45 (34.9)		
Marital status						3.339	0.347
Married	299 (96.5)	56 (98.2)	64 (98.5)	58 (98.3)	121 (93.8)		
Unmarried	11 (3.5)	1 (1.8)	1 (1.5)	1 (1.7)	8 (6.2)		
Education						25.036	**0** **.** **015**
Primary school and below	51 (16.5)	19 (33.3)	12 (18.5)	5 (8.5)	15 (11.6)		
Junior high school	82 (26.5)	15 (26.3)	18 (27.7)	14 (23.7)	35 (27.1)		
High school and equivalent	62 (20.0)	7 (12.3)	13 (20.0)	10 (16.9)	32 (24.8)		
Junior college	45 (14.5)	9 (15.8)	7 (10.8)	13 (22.0)	16 (12.4)		
University and above	70 (22.6)	7 (12.3)	15 (23.1)	17 (28.8)	31 (24.0)		
Occupation status						9.101	**0** **.** **028**
On duty	182 (58.7)	33 (57.9)	41 (63.1)	43 (72.9)	65 (50.4)		
Retirement	128 (41.3)	24 (42.1)	24 (36.9)	16 (27.1)	64 (49.6)		
Monthly income (yuan)						15.682	0.074
<3,000	67 (21.6)	15 (26.3)	15 (23.1)	11 (18.6)	26 (20.2)		
3,000–5,000	97 (31.3)	15 (26.3)	24 (36.9)	14 (23.7)	44 (34.1)		
5,000–10,000	83 (26.8)	15 (26.3)	12 (18.5)	14 (23.7)	42 (32.6)		
>10,000	63 (20.3)	12 (21.1)	14 (21.5)	20 (33.9)	17 (13.2)		
Classification of hypertension						6.322	0.388
Stage I	80 (25.8)	13 (22.8)	14 (21.5)	16 (27.1)	37 (28.7)		
Stage II	150 (48.4)	32 (56.1)	28 (43.1)	26 (44.1)	64 (49.6)		
Stage III	80 (25.8)	12 (21.1)	23 (35.4)	17 (28.8)	28 (21.7)		
Duration of hypertension (years)						72.514	**<0** **.** **001**
0.5–<1	69 (22.3)	3 (5.3)	15 (23.1)	35 (59.3)	16 (12.4)		
1–<5	101 (32.6)	17 (29.8)	21 (32.3)	17 (28.8)	46 (35.7)		
5–<10	74 (23.9)	22 (38.6)	16 (24.6)	2 (3.4)	34 (26.4)		
≥10	66 (21.3)	15 (26.3)	13 (20.0)	5 (8.5)	33 (25.6)		
Presence of comorbidity						32.557	**<0** **.** **001**
No	188 (60.6)	36 (63.2)	42 (64.6)	52 (88.1)	58 (45.0)		
Yes	122 (39.4)	21 (36.8)	23 (35.4)	7 (11.9)	71 (55.0)		
Residence						9.718	0.137
Urban area	206 (66.5)	32 (56.1)	38 (58.5)	42 (71.2)	94 (72.9)		
Rural area	47 (15.2)	9 (15.8)	12 (18.5)	7 (11.9)	19 (14.7)		
Others	57 (18.4)	16 (28.1)	15 (23.1)	10 (16.9)	16 (12.4)		
Type of medical insurance						25.167	**<0** **.** **001**
Employee medical insurance	183 (59.0)	18 (31.6)	41 (63.1)	38 (64.4)	86 (66.7)		
Resident medical insurance	58 (18.7)	21 (36.8)	8 (12.3)	9 (15.3)	20 (15.5)		
New rural cooperative medical scheme	69 (22.3)	18 (31.6)	16 (24.6)	12 (20.3)	23 (17.8)		
Medical institution for treatment						12.647	0.120
Grade A tertiary hospital	254 (81.9)	51 (89.5)	45 (69.2)	49 (83.1)	109 (84.5)		
Grade A secondary hospital	13 (4.2)	0 (0.0)	5 (7.7)	3 (5.1)	5 (3.9)		
Community hospital	38 (12.3)	6 (10.5)	13 (20.0)	7 (11.9)	12 (9.3)		
Others	5 (1.6)	0 (0.0)	2 (3.1)	0 (0.0)	3 (2.3)		
Smoking						1.647	0.649
No	243 (78.4)	45 (78.9)	48 (73.8)	45 (76.3)	105 (81.4)		
Yes	67 (21.6)	12 (21.1)	17 (26.2)	14 (23.7)	24 (18.6)		
Drinking alcohol						9.100	0.168
None	195 (62.9)	42 (73.7)	39 (60.0)	33 (55.9)	81 (62.8)		
Moderate	73 (23.5)	8 (14.0)	14 (21.5)	15 (25.4)	36 (27.9)		
Excessive	42 (13.5)	7 (12.3)	12 (18.5)	11 (18.6)	12 (9.3)		
Use of folic acid tablets						4.314	0.226
Yes	14 (4.5)	0 (0.0)	4 (6.2)	4 (6.8)	6 (4.7)		
No	296 (95.5)	57 (100.0)	61 (93.8)	55 (93.2)	123 (95.3)		
Use of B vitamins						4.627	0.197
Yes	16 (5.2)	0 (0.0)	4 (6.2)	3 (5.1)	9 (7.0)		
No	294 (94.8)	57 (100.0)	61 (93.8)	56 (94.9)	120 (93.0)		
BMI						12.029	0.061
Normal	58 (18.7)	5 (8.8)	14 (21.5)	11 (18.6)	28 (21.7)		
Overweight	149 (48.1)	26 (45.6)	34 (52.3)	23 (39.0)	66 (51.2)		
Obesity	103 (33.2)	26 (45.6)	17 (26.2)	25 (42.4)	35 (27.1)		
Hcy (*μ*mol/L)						7.711	0.198
10–<15	223 (71.9)	37 (64.9)	45 (69.2)	40 (67.8)	101 (78.3)		
15–<30	82 (26.5)	18 (31.6)	18 (27.7)	19 (32.2)	27 (20.9)		
≥30	5 (1.6)	2 (3.5)	2 (3.1)	0 (0.0)	1 (0.8)		
FBG						10.733	**0** **.** **013**
Normal	223 (71.9)	40 (70.2)	47 (72.3)	52 (88.1)	84 (65.1)		
Abnormal	87 (28.1)	17 (29.8)	18 (27.7)	7 (11.9)	45 (34.9)		
TG						6.385	0.094
Normal	214 (69.0)	33 (57.9)	51 (78.5)	39 (66.1)	91 (70.5)		
Abnormal	96 (31.0)	24 (42.1)	14 (21.5)	20 (33.9)	38 (29.5)		
TC						12.254	**0** **.** **007**
Normal	236 (76.1)	41 (71.9)	51 (78.5)	36 (61.0)	108 (83.7)		
Abnormal	74 (23.9)	16 (28.1)	14 (21.5)	23 (39.0)	21 (16.3)		
LDL-C						10.677	**0** **.** **014**
Normal	230 (74.2)	39 (68.4)	49 (75.4)	36 (61.0)	106 (82.2)		
Abnormal	80 (25.8)	18 (31.6)	16 (24.6)	23 (39.0)	23 (17.8)		
HDL-C						1.373	0.754
Normal	302 (97.4)	56 (98.2)	62 (95.4)	58 (98.3)	126 (97.7)		
Abnormal	8 (2.6)	1 (1.8)	3 (4.6)	1 (1.7)	3 (2.3)		
eHealth literacy	19.68 ± 10.70	15.28 ± 10.71	19.26 ± 9.37	19.86 ± 9.95	21.76 ± 11.17	5.060	**0** **.** **002**
Perceived social support	65.03 ± 11.04	62.30 ± 11.07	62.89 ± 9.73	62.78 ± 10.15	68.35 ± 11.30	7.070	**<0** **.** **001**
Perceived stress	20.64 ± 8.53	23.54 ± 9.65	22.06 ± 7.24	21.53 ± 8.45	18.22 ± 8.09	6.826	**<0** **.** **001**
Hospital anxiety	5.52 ± 3.46	6.16 ± 3.83	6.03 ± 2.84	6.24 ± 3.45	4.65 ± 3.43	4.855	**0** **.** **003**
Hospital depression	5.58 ± 3.93	7.02 ± 4.06	6.31 ± 4.23	5.98 ± 4.10	4.39 ± 3.28	8.402	**<0** **.** **001**
Psychological resilience	22.35 ± 4.37	21.30 ± 4.36	21.75 ± 3.85	21.66 ± 4.96	23.43 ± 4.15	4.809	**0** **.** **003**

BMI, body mass index; Hcy, plasma homocysteine; FBG, fasting blood glucose; TG, triglycerides; TC, total cholesterol; LDL-C, low-density lipoprotein cholesterol; HDL-C, high-density lipoprotein cholesterol; M, mean; SD, standard deviation. Categorical variables are presented as *n* (%) and continuous variables as M ± SD. Duration of hypertension was recorded as a continuous variable (years) and categorized only for descriptive presentation; the regression analysis used the continuous variable. Comparisons across profiles used one-way ANOVA for continuous variables and the chi-square test for categorical variables.

Bold values indicate statistically significant differences at *P* < 0.05.

### 3.3 Latent profiles of self-management behavior in patients with H-type hypertension

For latent profile analysis, the six dimensions of the self-management behavior scale were used as the explicit indicators, and models with 1–6 latent profiles were examined sequentially. The fit indices for each model are presented in Table 2. As the number of profiles increased, the AIC, BIC, and aBIC values decreased continuously. However, the LMR test results were not monotonic. Specifically, the LMR was non-significant for Model 3 (*P* = 0.100), significant for Model 4 (*P* = 0.006), and non-significant again for Model 5 (*P* = 0.532) and Model 6 (*P* = 0.926). The BLRT remained significant through Model 5. Given this discordance between the LMR and BLRT, the final model was not determined by a single criterion but by integrating multiple statistical and substantive considerations. Model 2 showed excellent entropy (0.947) and a significant LMR test, but it produced only a broad dichotomy that oversimplified the behavioral heterogeneity in this population and lacked the clinical granularity needed for targeted interventions. Model 3 yielded a substantially lower entropy (0.781) compared with Model 2, indicating poorer classification clarity, and the LMR test was not significant, suggesting that a 3-class solution did not significantly improve upon the 2-class model. Model 4 demonstrated a significant LMR test, confirming a statistically meaningful improvement over the 3-class model, along with better classification quality (entropy = 0.883) and clearly interpretable behavioral profiles. Model 5 showed a non-significant LMR test (*P* = 0.532), indicating that adding a fifth class did not significantly improve fit relative to Model 4. Although its entropy (0.892) was marginally higher than that of Model 4 (0.883), examination of the class proportions and class-specific means showed that the additional profile primarily subdivided the Low self-management profile (18.4% in Model 4) into two smaller profiles (8.4 and 10.6%), while the remaining major profiles retained highly similar proportions and response patterns. Moreover, this lower self-management region was further fragmented in the 6-class solution, suggesting limited stability of the additional subdivision. Therefore, the fifth profile was not considered to provide sufficient incremental substantive value to justify the additional model complexity. Model 6 also had a non-significant LMR test, and although each class exceeded the 5% threshold, the solution produced multiple small classes that were difficult to interpret clinically and theoretically and did not provide meaningful behavioral differentiation beyond Model 4. Therefore, considering the discordance between the LMR and BLRT, the continued decrease in information criteria, and the need for a solution that balanced statistical fit with clinical interpretability, parsimony, and model stability, Model 4 was ultimately selected as the optimal latent profile model. The class-specific means and profile plots for Models 2–6 are displayed in Supplementary Table S1 and Figure S1, respectively. To evaluate the classification accuracy and reliability of the LPA results, the average posterior probabilities for each of the four profiles were calculated. As shown in Table 3, the average posterior probabilities of correct classification for the four profiles ranged from 0.878 to 0.999, all exceeding the 0.80 criterion, indicating reliable classification for the 4-class model.

**Table 2:** Fit indices for the latent profile models of self-management behaviors in patients with H-type hypertension.

Model	Log-likelihood	AIC	BIC	aBIC	Entropy	*P*		Estimated class proportions
						LMR	BLRT	
1	−2,727.045	5,478.091	5,522.930	5,484.870	–	–	–	–
2	−2,534.898	5,107.795	5,178.790	5,118.529	0.947	<0.001	<0.001	0.235/0.765
3	−2,466.837	4,985.674	5,082.825	5,000.362	0.781	0.100	<0.001	0.342/0.200/0.458
4	−2,388.045	4,842.089	4,965.396	4,860.733	0.883	0.006	<0.001	0.184/0.210/0.190/0.416
5	−2,346.138	4,772.276	4,921.739	4,794.874	0.892	0.532	<0.001	0.084/0.206/0.190/0.106/0.413
6	−2,298.942	4,691.884	4,867.503	4,718.437	0.924	0.926	1.000	0.052/0.098/0.190/0.210/0.400/0.052

AIC = Akaike information criterion; BIC = Bayesian information criterion; aBIC = sample-size-adjusted BIC; LMR = Lo-Mendell-Rubin test; BLRT = bootstrap likelihood ratio test.

**Table 3:** Average posterior probabilities for the four latent profiles.

Model	Assigned latent profile			
	C1	C2	C3	C4
C1	0.999	0.000	0.001	0.000
C2	0.000	0.923	0.007	0.070
C3	0.002	0.024	0.954	0.020
C4	0.000	0.108	0.013	0.878

C1, low self-management pattern; C2, moderate self-management-treatment monitoring pattern; C3, moderate self-management-lifestyle pattern; C4, high self-management pattern.

Figure 1 displays the bias-adjusted profile plots based on the R3STEP procedure. The four profiles showed clearly differentiated patterns across the six self-management dimensions. The four profiles were labeled based on their distinctive score patterns. It should be noted that these profile labels (“Low”, “Moderate”, and “High”) are descriptive labels based on multivariate patterns across the six dimensions of the instrument, and are not equivalent to previously published standardized grading criteria (low: <60, moderate: 60–80, high: >80). The standardized classification reflects a unidimensional summary of overall self-management level based on the overall score, whereas the LPA profiles reflect distinct multidimensional behavioral configurations across the six self-management dimensions. Profile 1 had the lowest scores across all six dimensions of self-management and was therefore labeled the Low self-management pattern, comprising 18.4% of the sample. Profile 2 showed overall moderate scores on most dimensions but with relatively lower scores on the medication adherence and self-monitoring dimensions, and was labeled the Moderate self-management-treatment monitoring pattern, accounting for 21.0% of the sample. Profile 3 also showed moderate scores on most dimensions but had notably lower scores on the exercise management, activity & rest management, and emotion regulation dimensions. It was accordingly labeled the Moderate self-management-lifestyle pattern, representing 19.0% of the sample. Profile 4 exhibited relatively high scores on all dimensions and was named the High self-management pattern, accounting for 41.6% of the sample.

**Figure 1:**
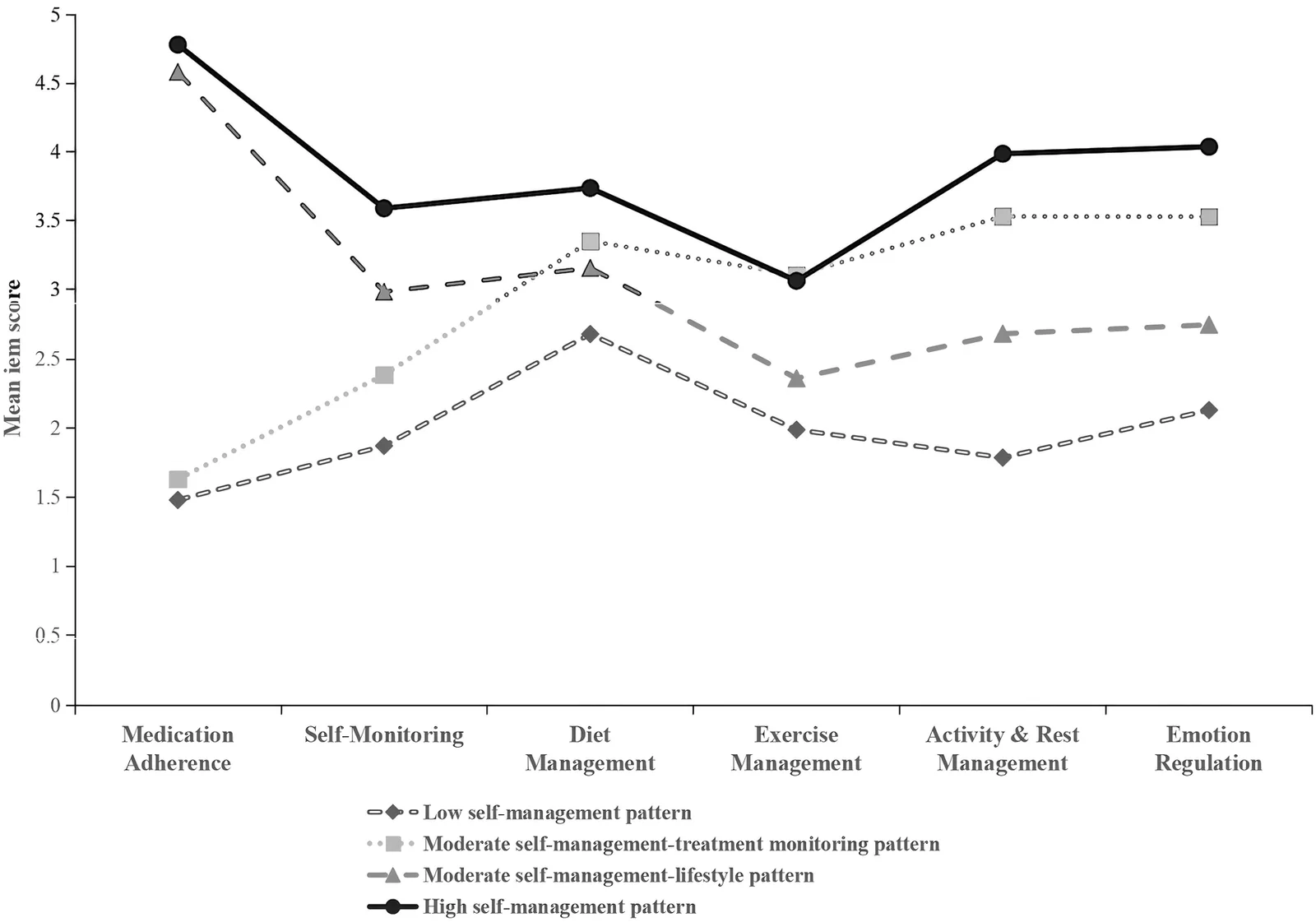
Distribution of self-management behaviors among patients with H-type hypertension across four profiles.

Table 1 presents the baseline characteristics of the four latent profiles across the 29 candidate variables examined. Univariate comparisons showed statistically significant differences across profiles in 15 of these variables (*P* < 0.05), which could be mapped onto the five levels of the Health Ecological Model, i.e., individual traits (age, education level, duration of hypertension, presence of comorbidity, FBG, TC, and LDL-C), behavioral characteristics (eHealth literacy, perceived stress, hospital anxiety and depression, and psychological resilience), interpersonal network (perceived social support), working and living conditions (occupation status), and policy environment (type of medical insurance). These comparisons are exploratory univariate findings and were not interpreted as independent associations in the final regression model.

### 3.4 Factors associated with latent profile membership

A multinomial logistic regression analysis using the R3STEP (robust three-step) approach was conducted to examine factors associated with latent profile membership, with the High self-management pattern set as the reference category. From 29 candidate variables spanning five ecological levels, seven core covariates were selected for entry into the multinomial logistic regression model based on the Health Ecological Model and prior empirical evidence (21–27). This approach ensured that each of the five ecological levels was represented by at least one available proxy variable while limiting model complexity in view of the available sample size and the size of the smallest profile (*n* = 57). Although the conventional 10-events-per-variable rule is commonly used as a heuristic for logistic regression, simulation evidence suggests that this threshold may be overly conservative under some conditions (51). For the psychosocial variables, anxiety was selected as the primary psychological distress indicator because of its strong and consistent association with self-management behaviors in hypertensive populations, and its specific relevance to treatment-monitoring behaviors (52). Perceived stress was excluded to avoid conceptual overlap with anxiety, while depression was excluded because its association with self-management in H-type hypertension is less established (52) and its inclusion alongside anxiety would raise concerns about discriminant validity. Psychological resilience was excluded because it is a protective resource whose effects are likely mediated through more proximal psychological states (24). The seven selected covariates are summarized in Table 4.

**Table 4:** Operationalization of the health ecological model: variable mapping and selection.

Ecological level	Candidate variables	Theoretical rationale for assignment	Selected for final model
Individual traits	Age, gender, education level, classification of hypertension, duration of hypertension, comorbidity, BMI, Hcy, FBG, TG, TC, LDL-C, HDL-C	These variables represent relatively stable demographic and clinical characteristics that reflect individuals’ social positioning, baseline health status, and physiological risk profile. These traits are relatively fixed or slowly changing over time and may be related to individuals’ physical capacity for self-management.	Duration of hypertension, comorbidity
Behavioral characteristics	Smoking, alcohol consumption, use of folic acid tablets, use of B vitamins, anxiety, depression, perceived stress, psychological resilience, eHealth literacy	These variables encompass modifiable health-related behaviors, treatment adherence practices, and psychological states that reflect current coping resources and digital skills relevant to engagement in self-management and are potentially amenable to intervention.	Anxiety, eHealth literacy
Interpersonal network	Marital status, perceived social support	These variables represent interpersonal resources and capture the availability of emotional, informational, and practical support from family and social networks that are relevant to self-management.	Perceived social support
Living and working conditions	Occupation status, monthly income, residence, medical institution for treatment	These variables reflect the structural context of individuals’ daily lives, including work-related demands, socioeconomic resources, geographic accessibility to healthcare, and patterns of healthcare utilization, which may be associated with the opportunities and constraints for engaging in self-management behaviors.	Occupation status
Policy and environment	Medical insurance	Medical insurance serves as a proxy for aspects of the broader policy and financing context, including healthcare access, financial protection, reimbursement, and out-of-pocket costs, which may be associated with treatment adherence, healthcare utilization, and self-management behaviors.	Medical insurance

BMI, body mass index; Hcy, plasma homocysteine; FBG, fasting blood glucose; TG, triglycerides; TC, total cholesterol; LDL-C, low-density lipoprotein cholesterol; HDL-C, high-density lipoprotein cholesterol.

Of the seven covariates, comorbidity, occupational status, and type of medical insurance were categorical variables requiring dummy coding. Specifically, comorbidity was coded as a binary variable (0 = absence, 1 = presence) with one dummy; occupation status was binary (on duty vs. retirement) with one dummy; and type of medical insurance was a three-category variable with two dummies (Employee medical insurance vs. New rural cooperative medical scheme; Resident medical insurance vs. New rural cooperative medical scheme). The remaining four covariates were entered as continuous variables. After accounting for dummy coding, eight covariate units were entered into the model. With four outcome classes, the model estimated three intercepts and 24 regression coefficients, yielding 27 estimated parameters. The EPV, calculated as the smallest class size (*n* = 57) divided by the number of predictor parameters (8), was approximately 7.1. Although this value was below the conventional rule of thumb of 10 EPV, simulation evidence suggests that acceptable model performance may still be achieved under some conditions with fewer than 10 EPV (51). Assessment of multicollinearity showed that all adjusted GVIF^[1/(2 × df)]^ values ranged from 1.038 to 1.121, well below the conventional threshold of 2.24 (equivalent to VIF < 5), indicating no severe multicollinearity. For type of medical insurance, the GVIF and adjusted GVIF values were 1.336 and 1.075, respectively. Tolerance values (1/VIF) for continuous and binary variables ranged from 0.796 to 0.927, all exceeding the conventional threshold of 0.10.

The R3STEP results indicated that comorbidity status, eHealth literacy, and perceived social support were significantly and independently associated with latent profile membership of self-management behaviors in patients with H-type hypertension. Specifically, compared with patients without comorbidity, those with comorbidity were significantly less likely to be classified into the Low self-management pattern (C1) relative to the High self-management pattern (C4) (*OR* = 0.067, 95% CI: 0.005–0.889, *P* = 0.041), and into the Moderate self-management-treatment monitoring pattern (C2) relative to C4 (*OR* = 0.240, 95% CI: 0.096–0.602, *P* *=* 0.002). No significant association was observed for the Moderate self-management-lifestyle pattern (C3) vs. C4 (OR = 0.534, 95% CI: 0.253–1.128, *P* = 0.102).

Higher eHealth literacy scores were associated with a lower likelihood of belonging to the Moderate self-management-lifestyle pattern (C3) relative to the High self-management pattern (C4) (*OR* = 0.927, 95% CI: 0.890–0.966, *P* < 0.001). No significant associations were observed for C1 vs. C4 or C2 vs. C4. Perceived social support was significantly associated with profile membership in two of the three comparisons. Higher perceived social support scores were associated with a lower likelihood of belonging to C2 vs. C4 (*OR* = 0.951, 95% CI: 0.911–0.993, *P* = 0.020), and C3 vs. C4 (*OR* = 0.942, 95% CI: 0.899–0.987, *P* = 0.010), but not for C1 vs. C4. By contrast, duration of hypertension, anxiety, type of medical insurance, and occupation status were not significantly associated with any of the profile comparisons. The detailed results are presented in Table 5.

**Table 5:** Results of multinomial logistic regression for factors associated with profile membership (R3STEP).

Variables		C1 vs. C4					C2 vs. C4					C3 vs. C4				
		OR	*β*	SE	95% CI	*P*	OR	*β*	SE	95% CI	*P*	OR	*β*	SE	95% CI	*P*
Comorbidity		**0** **.** **067**	−2.710	1.323	0.005–0.889	0.041	**0** **.** **240**	−1.428	0.470	0.096–0.602	0.002	0.534	−0.627	0.381	0.253–1.128	0.102
Duration of hypertension		0.301	−1.200	1.493	0.016–5.622	0.420	0.889	−0.118	0.074	0.769–1.027	0.091	0.964	−0.036	0.026	0.917–1.014	0.152
eHealth literacy		0.900	−0.106	0.080	0.768–1.053	0.185	0.981	−0.019	0.026	0.933–1.032	0.460	**0** **.** **927**	−0.075	0.021	0.890–0.966	**<0.001**
Perceived social support		0.919	−0.084	0.047	0.838–1.008	0.062	**0** **.** **951**	−0.050	0.022	0.911–0.993	0.020	**0** **.** **942**	−0.059	0.024	0.899–0.987	**0.010**
Hospital anxiety		1.314	0.273	0.172	0.937–1.842	0.112	1.132	0.124	0.068	0.991–1.293	0.068	1.050	0.049	0.057	0.940–1.173	0.400
Medical insurance	Employee medical insurance	0.438	−0.826	0.903	0.075–2.572	0.360	3.279	1.187	0.777	0.716–15.023	0.131	1.456	0.376	0.480	0.568–3.734	0.514
	Resident medical insurance	0.150	−1.899	2.633	0.001–26.103	0.470	1.855	0.618	0.882	0.329–10.450	0.481	2.355	0.857	0.570	0.771–7.199	0.130
Occupation status	Retirement	1.051	0.050	1.844	0.028–39.046	0.979	0.899	−0.106	0.545	0.309–2.619	0.837	0.447	−0.806	0.414	0.198–1.005	**0.052**

C4, High self-management pattern (reference category); C1, Low self-management pattern; C2, Moderate self-management-treatment monitoring pattern; C3, Moderate self-management-lifestyle pattern. Comorbidity: 0 = absence (reference), 1 = presence; Occupation status: 0 = on duty (reference), 1 = retirement; Medical insurance was dummy-coded with the New Rural Cooperative Medical Scheme as the reference category; OR, odds ratio; SE, standard error of the logit coefficient; 95% CI, 95% confidence interval of the odds ratio.

Bold values indicate statistically significant differences at *P* < 0.05.

## 4 Discussion

### 4.1 Heterogeneity of self-management behaviors in patients with H-type hypertension

Among patients with H-type hypertension, the mean standardized self-management score was 67.34 ± 13.82, corresponding to a moderate level of self-management based on the grading criteria of 60–80 points (46). This unidimensional summary, however, does not capture the substantial heterogeneity in behavioral patterns across the six subscales. Using a person-centered approach, LPA identified four distinct self-management profiles among patients with H-type hypertension, namely, Low self-management pattern, Moderate self-management-treatment monitoring pattern, Moderate self-management-lifestyle pattern, and High self-management pattern. These profiles reflect different configurations of behavioral strengths and weaknesses, offering a more nuanced understanding than the aggregate total score alone.

Profile 1, designated as the Low self-management pattern, comprised 18.4% of the sample and was characterized by the lowest scores across all six self-management dimensions. This group also exhibited the most unfavorable psychosocial profile, with the lowest levels of eHealth literacy, perceived social support, and psychological resilience, alongside the highest perceived stress. These findings were consistent with previous studies (9, 10, 53–55). Collectively, these patterns suggest that interventions targeting this subgroup might usefully prioritize stress management, health literacy enhancement, social support strengthening, and psychological resilience building.

Profile 2, designated as the Moderate self-management-treatment monitoring pattern, comprised 21.0% of the sample. Patients in this subgroup showed moderate scores across all six dimensions of self-management behaviors. However, their scores on medication adherence and self-monitoring were notably lower than those on other dimensions and than those observed in Profiles 3 and 4. This pattern suggests a selective deficit in the treatment-related components of hypertension management, despite relatively preserved lifestyle management (e.g., diet, exercise, and emotion regulation). Of note, this profile reflects general hypertension treatment-related behaviors (i.e., medication adherence and self-monitoring) and should not be interpreted as a validated H-type-specific phenotype, because the instrument used in the present study did not assess folic acid use, dietary folate, B-vitamin therapy, or homocysteine monitoring as validated domains. This profile is of particular clinical concern because medication adherence and regular blood pressure monitoring are fundamental to achieving hypertension control (8, 56). In H-type hypertension, homocysteine-lowering therapy, including folic acid or B-vitamin supplementation, may be considered according to individual clinical assessment and treatment indication, in addition to antihypertensive agents. Because perceived health needs, symptom experience, medication complexity, and polypharmacy were not measured in the present study, the reasons for this selective deficit can only be hypothesized. One possible explanation is that patients in this subgroup do not perceive an immediate need for treatment monitoring, because they experience few symptoms or have limited awareness of the asymptomatic nature of hypertension and its long-term risks. Another possibility is that treatment burden, such as polypharmacy or complex dosing schedules, undermines adherence even when patients are motivated to manage their lifestyle. These hypotheses warrant further investigation. If confirmed, patients in this subgroup could benefit from targeted interventions focusing on simplifying medication regimens, utilizing digital reminders, and enhancing self-monitoring skills through telemonitoring or regular feedback from healthcare providers.

Profile 3, designated as the Moderate self-management-lifestyle pattern, accounted for 19.0% of the sample. Participants in this subgroup demonstrated moderate scores across most self-management dimensions, but exhibited notably lower scores on lifestyle-related behaviors, particularly exercise, activity & rest, and emotion regulation. Although their medication adherence and self-monitoring scores were relatively preserved compared with Profile 2, their deficits in lifestyle behaviors suggest a distinct pattern of self-management challenges that are rooted in daily behavioral routines rather than treatment adherence. This pattern is clinically significant given the well-established evidence that lifestyle modification, especially regular physical activity, is a cornerstone of hypertension management. Research (57) shows that exercise plays a pivotal role in blood pressure control among older hypertensive patients, with regular physical activity contributing to blood pressure control and complication prevention (58, 59). Interventions tailored to this subgroup might therefore prioritize lifestyle modification, with a particular emphasis on establishing sustainable exercise routines.

Profile 4, designated as the High self-management pattern, was the largest subgroup, comprising 41.6% of the sample. Participants in this profile demonstrated the highest scores across all self-management dimensions, indicating a generally favorable behavioral profile. However, even within this relatively well-managed group, exercise management emerged as a relative weakness, which may be related to the finding that 78.3% of patients in this subgroup were overweight or obese. Given that these patients already reported good medication adherence and monitoring, interventions for this subgroup could focus on the specific deficit in exercise behavior. Structured lifestyle education combined with self-monitoring, regular feedback, and coping-skills support has been shown to improve blood pressure control (56), and personalized, interest-based exercise plans with structured guidance may help sustain exercise adherence in this group. Future research should evaluate whether such targeted interventions in this subgroup can yield meaningful improvements in exercise adherence.

### 4.2 Factors associated with self-management profile membership in patients with H-type hypertension

At the individual-trait level, comorbidity status was significantly associated with latent profile membership. Specifically, patients without comorbidity tended to fall into either the Low self-management pattern or the Moderate self-management-treatment monitoring pattern, rather than the High self-management pattern. Consistently, AlHadlaq et al. (60) suggested that patients with cardiovascular-related chronic conditions often demonstrate higher levels of self-management behavior. One possible explanation is that patients without comorbidity may have fewer routine healthcare contacts and therefore less exposure to professional guidance and health education, which could contribute to lower engagement in self-management behaviors. However, several alternative explanations are equally plausible. First, patients with more comorbidities may have greater disease severity and treatment burden, which may paradoxically heighten their health awareness and motivate more proactive disease management. Second, healthcare utilization patterns may differ between those with and without comorbidity, with the former having more frequent interactions with providers that reinforce self-management skills. Third, age differences may confound this association, as older patients with more comorbidities may receive more family and social support. Fourth, health awareness and health literacy may vary systematically between groups, independent of the presence of comorbidity. Given the cross-sectional design, the present data cannot adjudicate between these competing explanations. This finding should therefore be interpreted as hypothesis-generating, and future longitudinal studies with detailed measures of healthcare utilization, health literacy, and treatment burden are needed to disentangle the mechanisms underlying this association. Of note, because patients with serious comorbidities specified in the exclusion criteria were excluded at recruitment, the comorbidity variable in this study contrasts patients with no comorbidity against those with milder, well-compensated conditions. The observed relationship between comorbidity and self-management profile may therefore be underestimated in the broader H-type hypertension population.

At the behavioral characteristics level, higher eHealth literacy was associated with membership in the High self-management pattern, whereas lower eHealth literacy was more common among patients in the Moderate self-management-lifestyle pattern. This finding is consistent with evidence linking eHealth literacy to health-related behaviors among patients with hypertension. Higher eHealth literacy may enhance patients’ ability to access, understand, evaluate, and use digital health information and services (61). Previous research has also identified higher eHealth literacy as being associated with better medication adherence among patients with hypertension (62). Conversely, limited health literacy may create barriers to the effective use of online health information and eHealth services (61). These findings suggest that healthcare providers could consider patients’ eHealth literacy when providing self-management support and offer accessible, credible, and literacy-sensitive digital health resources.

At the interpersonal network level, greater perceived social support was significantly associated with membership in the High self-management pattern. This finding aligns with prior research (9, 10), in which greater social support was related to better self-management behaviors in patients with H-type hypertension. Perceived social support reflects an individual's perception of the availability of emotional, informational, and practical assistance from family, friends, and broader social networks. Previous research has linked higher perceived social support with better blood pressure control among patients with hypertension (25). In addition, the association between perceived social support and profile membership was more pronounced for the Moderate self-management profiles than for the Low self-management profile, for which the association did not reach statistical significance. This observed gradient effect may suggest that social support is more strongly associated with profile membership when patients already possess a baseline level of self-management capacity. For those with lower self-management, additional structural or psychological barriers may need to be addressed before social support can be effectively leveraged. For this reason, healthcare providers may consider assessing patients’ available support resources and, where appropriate, involving family members in chronic disease management and connecting patients with suitable peer-support resources (63–65).

It is noteworthy that, among the seven covariates preselected based on the Health Ecological Model, only comorbidity status (individual trait level), eHealth literacy (behavioral characteristics level), and perceived social support (interpersonal network level) emerged as significant independent correlates of profile membership in the multivariable analysis. In contrast, variables representing the working and living conditions level (occupational status) and the policy environment level (type of medical insurance), as well as hypertension duration and anxiety, did not reach statistical significance. Several explanations may account for this result. First, the non-significance of distal-level variables in the multivariable model does not necessarily indicate that these factors are clinically unimportant. Rather, one possible explanation that was not directly tested in this study is that the associations between these proxy variables and profile membership may operate through more proximal factors. For example, it is possible that type of medical insurance is related to self-management partly through its association with healthcare access and financial burden, which in turn may be related to patients’ health literacy, perceived support, and psychological well-being. Similarly, the association between occupational status and self-management might be partly explained by differences in time availability, stress exposure, and social networks, which are more proximally related to daily behavioral routines. These mediation pathways remain hypothetical and require future testing. Second, the relatively modest sample size (particularly the smallest profile *n* = 57) limited the statistical power to detect modest but potentially meaningful effects from these distal variables. Third, the cross-sectional design precludes examination of the temporal pathways through which distal ecological factors may be related to self-management over time. Regarding anxiety, it did not reach statistical significance in the model. However, because anxiety and self-management may be bidirectionally related, a brief hypothesis-generating discussion is warranted. It is possible that anxiety is associated with less consistent treatment monitoring, for example by reducing adherence to blood pressure measurement or prompting avoidance of unfavorable results (66), and it is equally possible that poor disease control exacerbates anxiety. Given the cross-sectional design, this interpretation is speculative and requires testing in future longitudinal studies.

Taken together, among the variables available in this study, proximal individual- and interpersonal-level factors showed more pronounced associations with profile membership than the selected proxy variables representing distal ecological levels. This does not diminish the importance of the broader ecological context. Instead, these findings suggest that interventions targeting modifiable proximal factors may hold greater potential for improving self-management and therefore warrant prospective evaluation, while broader distal ecological factors, such as policy- and environment-level changes, should also be considered.

## 5 Limitations

Several limitations should be acknowledged. First, the cross-sectional design precludes causal inference, and all reported relationships are associations observed at a single time point. Longitudinal designs with latent transition analysis are needed to establish temporal ordering and profile transitions. Second, participants were recruited by convenience sampling from a single tertiary hospital in Shandong Province, and 81.9% of the sample reported a tertiary hospital as their usual source of care. Consequently, the profiles and their prevalence may not generalize to primary care or rural community settings. In addition, patients with serious cardiac, cerebrovascular, pulmonary, and renal disease were excluded. This likely rendered the sample healthier than the broader H-type hypertension population and may have compressed the range of self-management behaviors. It may also have influenced the observed relationship between comorbidity and profile membership, which should therefore be interpreted as applicable to patients with well-compensated comorbidities. Third, the behavioral indicators relied on self-reported measures, which are subject to recall and social desirability biases and may not fully capture objective behavioral patterns. The Harman single-factor test did not suggest a dominant general factor, but this test is a weak diagnostic and cannot rule out common method variance. Fourth, several potential confounders were unavailable, and residual confounding cannot be excluded. Although a single baseline blood pressure measurement was obtained at recruitment, longitudinal blood pressure control status, the specific antihypertensive regimen, medication burden, prior cardiovascular disease, renal function, and objective measures of treatment adherence were not available for analysis. Fifth, the self-management instrument was designed for general hypertension and does not capture H-type-specific behaviors (e.g., folic acid/B-vitamin supplementation, dietary folate, homocysteine retesting). As no validated instrument specifically designed to measure self-management behaviors unique to H-type hypertension was identified, the profiles reflect general self-management patterns rather than disease-specific profiles. The development and psychometric validation of a dedicated H-type hypertension self-management instrument is warranted in future research. Sixth, although the sample size (*n* = 310) is within the range considered workable in previous LPA simulation studies, it remains below the more conservative sample size of approximately 500 suggested in methodological reviews for reliable class enumeration under many conditions (47). The smallest identified profile contained 57 participants, indicating that no profile was extremely sparse. Additionally, with seven covariates (EPV ≈ 7.1), the multinomial regression model had a modest EPV, and extreme odds ratios should therefore be interpreted cautiously and warrant replication. Finally, the profiles were not externally validated, and no longitudinal cardiovascular outcomes were assessed. Their prognostic significance, therefore, remains unknown. Future multicenter, community-based cohorts with larger samples are needed to externally validate these profiles, examine their predictive value for hard clinical endpoints (e.g., sustained blood pressure control, target organ damage, cardiovascular events, renal progression, hospitalization, and mortality), and determine whether profile-based classification can inform individualized management strategies.

## 6 Conclusion

The study identified heterogeneity in self-management behaviors among patients with H-type hypertension. Latent profile analysis revealed four descriptively distinct profiles, i.e., Low self-management pattern, Moderate self-management-treatment monitoring pattern, Moderate self-management-lifestyle pattern, and High self-management pattern. Profile membership was associated with comorbidity status, eHealth literacy, and perceived social support, whereas variables representing working and living conditions and the policy environment did not reach statistical significance. These findings suggest that, within the Health Ecological Model framework, proximal individual- and interpersonal-level factors may be more directly associated with self-management profiles in this population than distal structural factors. Accordingly, healthcare professionals, including nurses, could prioritize interventions targeting modifiable proximal factors such as eHealth literacy enhancement, social support strengthening, and comorbidity-tailored education. However, given the cross-sectional design, the effectiveness of profile-tailored strategies requires confirmation in future prospective and intervention studies.

## Data Availability

The original contributions presented in the study are included in the article/Supplementary Material, further inquiries can be directed to the corresponding author.
