# Supplementary material for: Self-management behaviors in patients with H-type hypertension: a latent profile analysis within the health ecological model
Source: Front Cardiovasc Med. 2026 Oct 21;13:1903891. doi: 10.3389/fcvm.2026.1903891 (PMC13731399; doi:10.3389/fcvm.2026.1903891)
Supplement: Supplementary file 1 [file Datasheet1.pdf]

Supplementary Table S1. Class-specific means for Models 2–6

Model	Latent Class	Medication Adherence	Self-Monitoring	Diet Management	Exercise Management	Activity & Rest Management	Emotion Regulation
2	1	1.548	2.181	3.057	2.647	2.816	3.006
	2	4.691	3.343	3.536	2.809	3.486	3.535
3	1	4.410	2.875	3.119	2.334	2.817	2.828
	2	1.372	2.145	3.092	2.655	2.849	3.042
	3	4.800	3.633	3.795	3.140	3.917	4.000
4	1	1.477	1.869	2.679	1.986	1.784	2.128
	2	1.628	2.383	3.347	3.100	3.527	3.525
	3	4.580	2.977	3.151	2.358	2.681	2.746
	4	4.780	3.586	3.734	3.057	3.984	4.035
5	1	1.286	1.868	2.809	2.094	1.976	2.372
	2	1.152	2.356	3.373	3.142	3.582	3.640
	3	3.546	2.761	3.345	2.695	3.198	3.231
	4	4.922	3.036	3.077	2.276	2.683	2.552
	5	4.945	3.676	3.777	3.107	3.941	4.086
6	1	1.001	1.444	2.575	1.850	1.734	2.139
	2	1.001	2.275	3.358	2.939	3.455	3.581
	3	2.091	2.651	3.183	2.914	2.880	3.010
	4	3.638	2.774	3.352	2.674	3.183	3.207
	5	4.955	3.050	3.087	2.298	2.719	2.598
	6	4.965	3.698	3.788	3.120	3.947	4.101

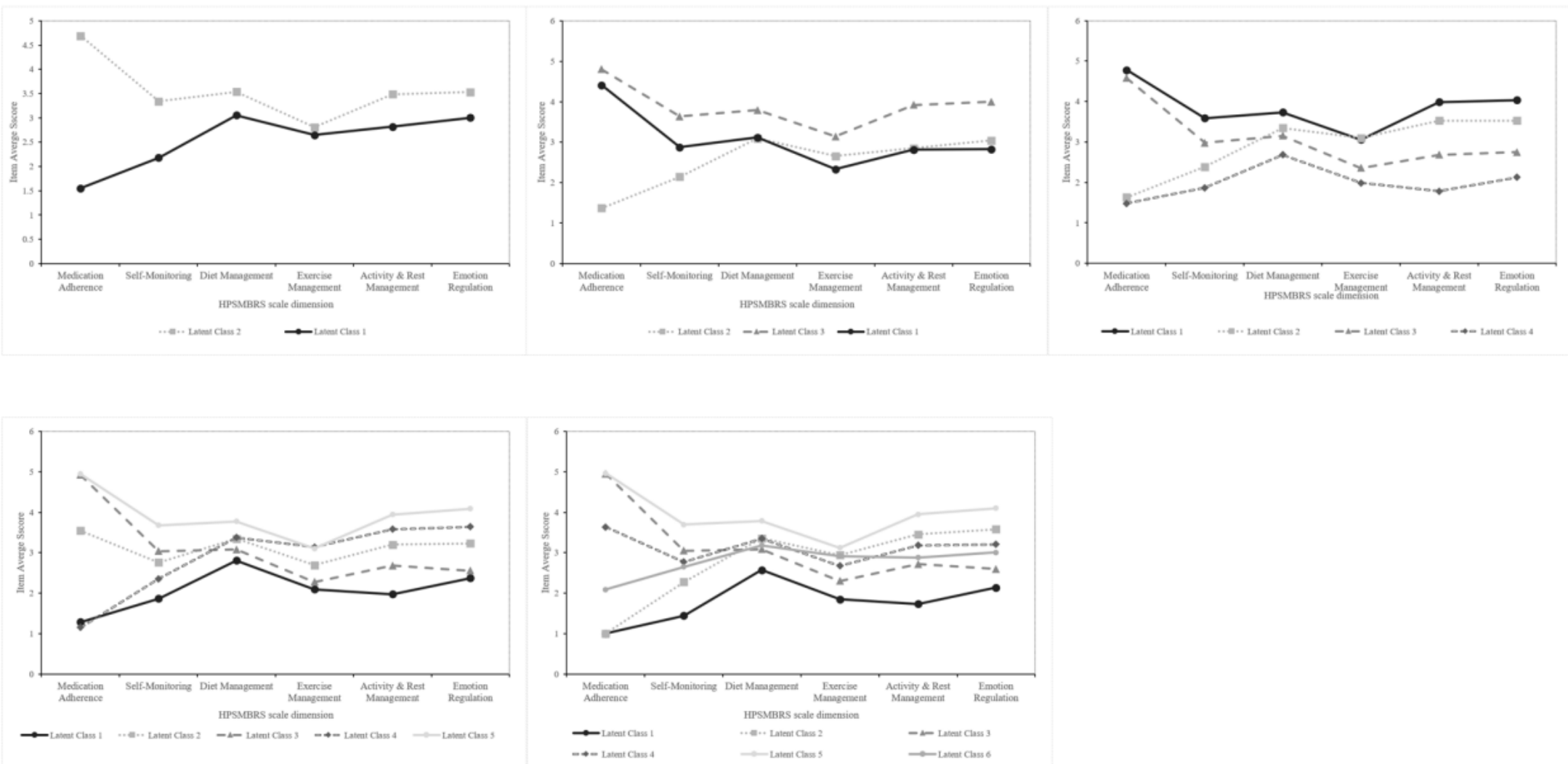

Supplementary Figure S1. Profile plots for Models 2–6
